# Supplementary material for: The Development of Toxoplasma gondii Recombinant Trivalent Chimeric Proteins as an Alternative to Toxoplasma Lysate Antigen (TLA) in Enzyme-Linked Immunosorbent Assay (ELISA) for the Detection of Immunoglobulin G (IgG) in Small Ruminants
Source: Int J Mol Sci. 2024 Apr 16;25(8):4384. doi: 10.3390/ijms25084384 (PMC11049947; doi:10.3390/ijms25084384)
Supplement: Supplementary file 1 [file ijms-25-04384-s001.zip › Figure S3.pdf]

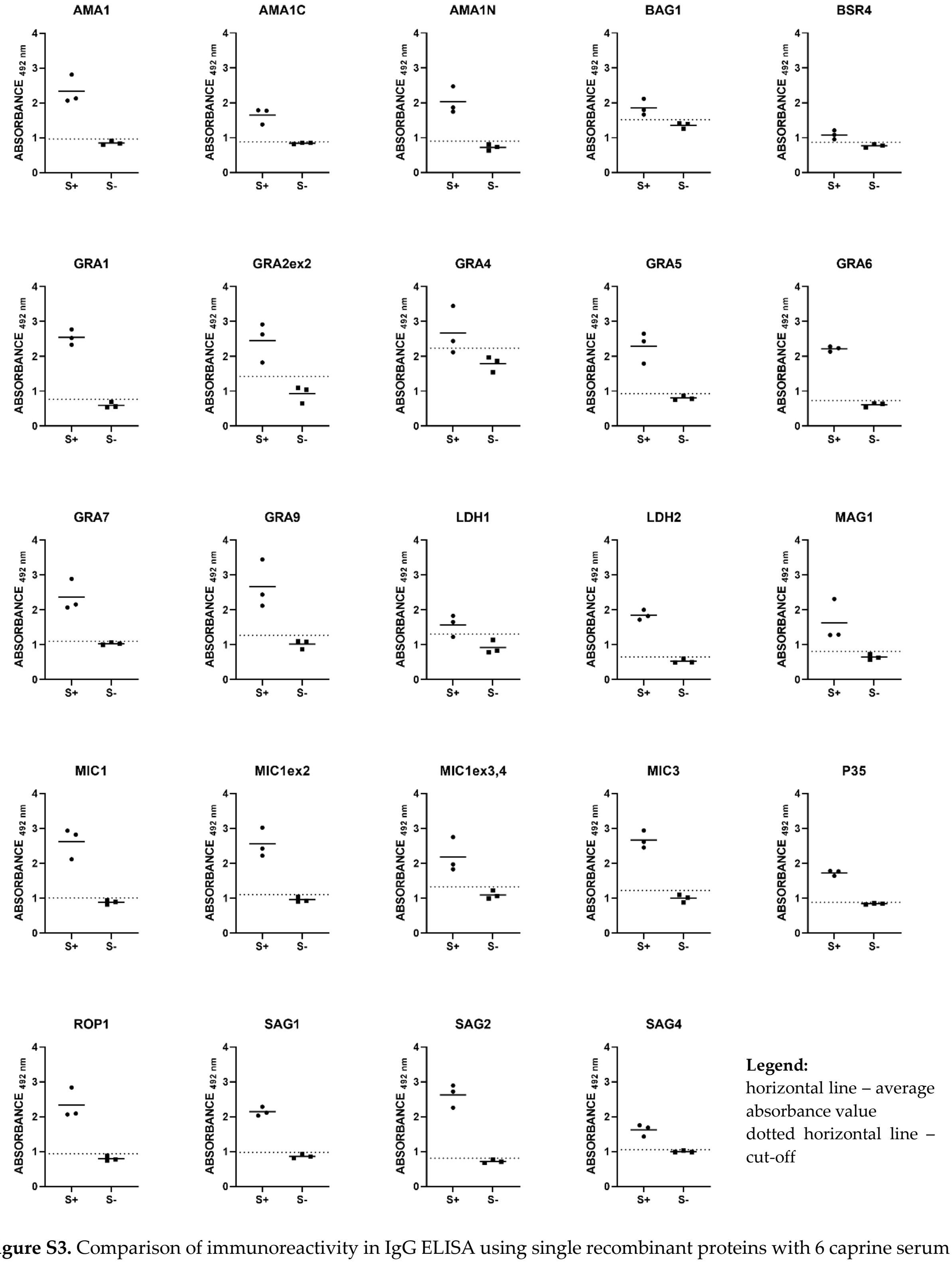

Figure S3. Comparison of immunoreactivity in IgG ELISA using single recombinant proteins with 6 caprine serum samples: three from naturally infected goats (S+) and three from seronegative animals (S-). The secondary antibody dilution used was 1:16 000.
