# Supplementary material for: The Development of Toxoplasma gondii Recombinant Trivalent Chimeric Proteins as an Alternative to Toxoplasma Lysate Antigen (TLA) in Enzyme-Linked Immunosorbent Assay (ELISA) for the Detection of Immunoglobulin G (IgG) in Small Ruminants
Source: Int J Mol Sci. 2024 Apr 16;25(8):4384. doi: 10.3390/ijms25084384 (PMC11049947; doi:10.3390/ijms25084384)
Supplement: Supplementary file 1 [file ijms-25-04384-s001.zip › Figure S5.pdf]

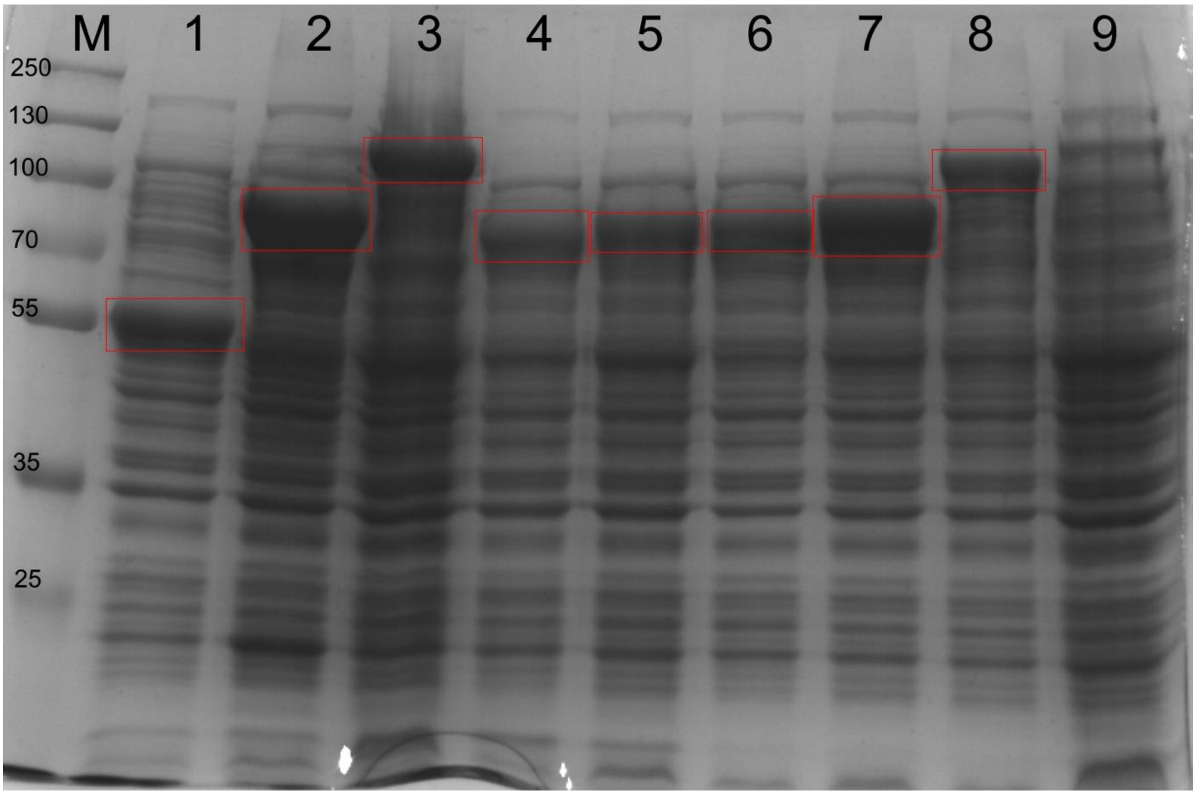

Figure S5. Coomassie blue-stained SDS-PAGE (10% polyacrylamide gel) separation of protein lysates.

Lines:

M – PageRuler™ Plus Prestained Protein Ladder (Thermo Fisher Scientific, Waltham, MA, USA)
1 – *E. coli* Rosetta(DE3)pLysS+pET30/SAG1-SAG2 cell lysate
2 – *E. coli* Rosetta(DE3)pLysS+pET30/SAG1-SAG2-MIC3 cell lysate
3 – *E. coli* Rosetta(DE3)pLysS+pET30/SAG1-SAG2-AMA1 cell lysate
4 – *E. coli* Rosetta(DE3)pLysS+pET30/SAG1-SAG2-GRA1 cell lysate
5 – *E. coli* Rosetta(DE3)pLysS+pET30/SAG1-SAG2-GRA6 cell lysate
6 – *E. coli* Rosetta(DE3)pLysS+pET30/SAG1-SAG2-GRA7 cell lysate
7 – *E. coli* Rosetta(DE3)pLysS+pET30/SAG1-SAG2-GRA9 cell lysate
8 – *E. coli* Rosetta(DE3)pLysS+pET30/SAG1-SAG2-MIC1 cell lysate
9 – *E. coli* Rosetta(DE3)pLysS+pET30 Ek/LIC cell lysate (negative control)
