# Supplementary material for: The Development of Toxoplasma gondii Recombinant Trivalent Chimeric Proteins as an Alternative to Toxoplasma Lysate Antigen (TLA) in Enzyme-Linked Immunosorbent Assay (ELISA) for the Detection of Immunoglobulin G (IgG) in Small Ruminants
Source: Int J Mol Sci. 2024 Apr 16;25(8):4384. doi: 10.3390/ijms25084384 (PMC11049947; doi:10.3390/ijms25084384)
Supplement: Supplementary file 1 [file ijms-25-04384-s001.zip › Figure S6.pdf]

Figure S6. Amino acid sequences of recombinant chimeric proteins

SAG1-SAG2(SAG1 49-310 AA; SAG2 30-170 AA)

```
  1 MHHHHHHSSG LVPRGSGMKE TAAAKFERQH MDSPDPDPPL VANQVVTCPD KKSTAAVILT
 61 PTENHFTLKC PKTALTEPPT LAYSPNRQIC PAGTTSSCTS KAVTLSSLIP EAEDSWWTGD
121 SASLDTAGIK LTVPIEKFPV TTQTFVVGCI KGDDAQSCMV TVTVQARASS VVNNVARCSY
181 GADSTLGPVK LSAEGPTTMT LVCGKDGVKV PQDNNQYCSG TTLTGCNEKS FKDILPKLTE
241 NPWQGNASSD KGATLTIKKE AFPAESKSVI IGCTGGSPEK HHCTVKLEFA GAAGSAKSAE
301 TPAPIECTAG ATKTVDAPSS GSVVFQCGDK LTISPSGEGD VFYGKECTDS RKLTTVLPGA
361 VLTAKVQQPA KGPATYTLSY DGTPEKPQVL CYKCVAEAGA PAGRNNDGSS APTPKDCKLI
421 VRVPGADGRV TSGFDPVSLT DLGTDDDDKS PGFSSTMAIS DPNSSSVDKL AAALEHHHHH
481 H
```

SAG1-SAG2-AMA1 (SAG1 49-310 AA; SAG2 30-170 AA; AMA1 67-568 AA)

```
  1 MHHHHHHSSG LVPRGSGMKE TAAAKFERQH MDSPDPDPPL VANQVVTCPD KKSTAAVILT
 61 PTENHFTLKC PKTALTEPPT LAYSPNRQIC PAGTTSSCTS KAVTLSSLIP EAEDSWWTGD
121 SASLDTAGIK LTVPIEKFPV TTQTFVVGCI KGDDAQSCMV TVTVQARASS VVNNVARCSY
181 GADSTLGPVK LSAEGPTTMT LVCGKDGVKV PQDNNQYCSG TTLTGCNEKS FKDILPKLTE
241 NPWQGNASSD KGATLTIKKE AFPAESKSVI IGCTGGSPEK HHCTVKLEFA GAAGSAKSAE
301 TPAPIECTAG ATKTVDAPSS GSVVFQCGDK LTISPSGEGD VFYGKECTDS RKLTTVLPGA
361 VLTAKVQQPA KGPATYTLSY DGTPEKPQVL CYKCVAEAGA PAGRNNDGSS APTPKDCKLI
421 VRVPGADGRV TSGFDPVSLT DLGTDDDDKS PGFSSTMAIT SGNPFQANVE MKTFMERFNL
481 THHHQSGIYV DLGQDKEVDG TLYREPAGLC PIWGKHIELQ QPDRPPYRNN FLEDVPTEKE
541 YKQSGNPLPG GFNLNFVTPS GQRISPFPME LLEKNSNIKA STDLGRCAEF AFKTVAMDKN
601 NKATKYRYPF VYDSKKRLCH ILYVSMQLME GKKYCSVKGE PPDLTWYCFK PRKSVTENHH
661 LIYGSAYVGE NPDAFISKCP NQALRGYRFG VWKKGRCLDY TELTDTVIER VESKAQCWVK
721 TFENDGVASD QPHTYPLTSQ ASWNDWWPLH QSDQPHSGGV GRNYGFYYVD TTGEGKCALS
781 DQVPDCLVSD SAAVSYTAAG SLSEETPNFI IPSNPSVTPP TPETALQCTA DKFPDSFGAC
841 DVQACKRQKT SCVGGQIQST SVDCTADEQN ECGSNTALIA GLAVGGVLLL ALLGGGCYFA
901 KRLDRNKGVQ AAHHEHEFQS DRGARKKRPS DLMQEAEPSF WDEAEENIEQ DGETHVMVEG
961 ESDPNSSSVD KLAAALEHHH HHH
```

SAG1-SAG2-AMA1S (SAG1 49-310 AA; SAG2 30-170 AA; AMA1N 67-483 AA)

```
  1 MHHHHHHSSG LVPRGSGMKE TAAAKFERQH MDSPDPDPPL VANQVVTCPD KKSTAAVILT
 61 PTENHFTLKC PKTALTEPPT LAYSPNRQIC PAGTTSSCTS KAVTLSSLIP EAEDSWWTGD
121 SASLDTAGIK LTVPIEKFPV TTQTFVVGCI KGDDAQSCMV TVTVQARASS VVNNVARCSY
181 GADSTLGPVK LSAEGPTTMT LVCGKDGVKV PQDNNQYCSG TTLTGCNEKS FKDILPKLTE
241 NPWQGNASSD KGATLTIKKE AFPAESKSVI IGCTGGSPEK HHCTVKLEFA GAAGSAKSAE
301 TPAPIECTAG ATKTVDAPSS GSVVFQCGDK LTISPSGEGD VFYGKECTDS RKLTTVLPGA
361 VLTAKVQQPA KGPATYTLSY DGTPEKPQVL CYKCVAEAGA PAGRNNDGSS APTPKDCKLI
421 VRVPGADGRV TSGFDPVSLT TSGNPFQANV EMKTFMERFN LTHHHQSGIY VDLGQDKEVD
481 GTLYREPAGL CPIWGKHIEL QQPDRPPYRN NFLEDVPTEK EYKQSGNPLP GGFNLNFVTP
541 SGQRISPFPM ELLEKNSNIK ASTDLGRCAE FAFKTVAMDK NNKATKYRYP FVYDSKKRLC
601 HILYVSMQLM EGKKYCSVKG EPPDLTWYCF KPRKSVTENH HLIYGSAYVG ENPDAFISKC
661 PNQALRGYRF GVWKKGRCLD YTELTDTVIE RVESKAQCWV KTFENDGVAS DQPHTYPLTS
721 QASWNDWWPL HQSDQPHSGG VGRNYGFYYV DTTGEGKCAL SDQVPDCLVS DSAAVSYTAA
781 GSLSEETPNF IIPSNPSVTP PTPETALQCT ADKFPDSFGA CDVQACKRQK TSCVGGQIQS
841 TSVDCTADEQ NECGSNTDLG TDDDDKSPGF SSTMAISDPN SSSVDKLAAA LEHHHHHH
```

SAG1-SAG2-GRA1 (SAG1 49-310 AA; SAG2 30-170 AA; GRA1 24-190 AA)

```
  1 MHHHHHHSSG LVPRGSGMKE TAAAKFERQH MDSPDPDPPL VANQVVTCPD KKSTAAVILT
 61 PTENHFTLKC PKTALTEPPT LAYSPNRQIC PAGTTSSCTS KAVTLSSLIP EAEDSWWTGD
121 SASLDTAGIK LTVPIEKFPV TTQTFVVGCI KGDDAQSCMV TVTVQARASS VVNNVARCSY
181 GADSTLGPVK LSAEGPTTMT LVCGKDGVKV PQDNNQYCSG TTLTGCNEKS FKDILPKLTE
241 NPWQGNASSD KGATLTIKKE AFPAESKSVI IGCTGGSPEK HHCTVKLEFA GAAGSAKSAE
```

301 TPAPIECTAG ATKTVDAPSS GSVVFQCGDK LTISPSGEGD VFYGKECTDS RKLTTVLPGA
361 VLTAKVQQPA KGPATYTLSY DGTPEKPQVL CYKCVAEAGA PAGRNNDGSS APTPKDCKLI
421 VRVPGADGRV TSGFDPVSLT DLGTDDDDKS PGFSSTMAIA AEGGDNQSSA VSDRASLFGL
481 LSGGTGQGLG IGESVDLEMM GNTYRVERPT GNPDLLKIAI KASDGSYSEV GNVNVEEVID
541 TMKSMQRDED IFLRALNKGE TVEEAIEDVA QAEGLNSEQT LQLEDAVSAV ASVVQDEMKV
601 IDDVQQLEKD KQQLKDDIGF LTGERESDPN SSSVDKLAAA LEHHHHHH

SAG1-SAG2-GRA2 (SAG1 49-310 AA; SAG2 30-170 AA; GRA2 51-185 AA)

1 MHHHHHHSSG LVPRGSGMKE TAAAKFERQH MDSPDPDPPL VANQVVTCPD KKSTAAVILT
61 PTENHFTLKC PKTALTEPPT LAYSPNRQIC PAGTTSSCTS KAVTLSSLIP EAEDSWWTGD
121 SASLDTAGIK LTVPIEKFPV TTQTFVVGCI KGDDAQSCMV TVTVQARASS VVNNVARCSY
181 GADSTLGPVK LSAEGPTTMT LVCGKDGVKV PQDNNQYCSG TTLTGCNEKS FKDILPKLTE
241 NPWQGNASSD KGATLTIKKE AFPAESKSVI IGCTGGSPEK HHCTVKLEFA GAAGSAKSAE
301 TPAPIECTAG ATKTVDAPSS GSVVFQCGDK LTISPSGEGD VFYGKECTDS RKLTTVLPGA
361 VLTAKVQQPA KGPATYTLSY DGTPEKPQVL CYKCVAEAGA PAGRNNDGSS APTPKDCKLI
421 VRVPGADGRV TSGFDPVSLT GKGEHTPPLP DERQQEPEEP VSQRASRVAE QLFRKFLKFA
481 ENVGHHSEKA FKKAKVVAEK GFTAAKTHTV RGFKVAKEAA GRGMVTVGKK LANVESDRST
541 TTTQAPDSPN GLAETEVPVE PQQRAAHVPV PDFSQDLGTD DDDKSPGFSS TMAISDPNSS
601 SVDKLAAALE HHHHHH

SAG1-SAG2-GRA5 (SAG1 49-310 AA; SAG2 30-170 AA; GRA5 26-120 AA)

1 MHHHHHHSSG LVPRGSGMKE TAAAKFERQH MDSPDPDPPL VANQVVTCPD KKSTAAVILT
61 PTENHFTLKC PKTALTEPPT LAYSPNRQIC PAGTTSSCTS KAVTLSSLIP EAEDSWWTGD
121 SASLDTAGIK LTVPIEKFPV TTQTFVVGCI KGDDAQSCMV TVTVQARASS VVNNVARCSY
181 GADSTLGPVK LSAEGPTTMT LVCGKDGVKV PQDNNQYCSG TTLTGCNEKS FKDILPKLTE
241 NPWQGNASSD KGATLTIKKE AFPAESKSVI IGCTGGSPEK HHCTVKLEFA GAAGSAKSAE
301 TPAPIECTAG ATKTVDAPSS GSVVFQCGDK LTISPSGEGD VFYGKECTDS RKLTTVLPGA
361 VLTAKVQQPA KGPATYTLSY DGTPEKPQVL CYKCVAEAGA PAGRNNDGSS APTPKDCKLI
421 VRVPGADGRV TSGFDPVSLT DLGTDDDDKS PGFSSTMAIG STRDVGSGGD DSEGARGREQ
481 QQVQQHEQNE DRSLFERGRA AVTGHPVRTA VGLAAAVVAV VSLLRLLKRR RRRAIQEESK
541 ESATAEEEEV AEEESDPNSS SVDKLAAALE HHHHHH

SAG1-SAG2-GRA6 (SAG1 49-310 AA; SAG2 30-170 AA; GRA6 30-228 AA)

1 MHHHHHHSSG LVPRGSGMKE TAAAKFERQH MDSPDPDPPL VANQVVTCPD KKSTAAVILT
61 PTENHFTLKC PKTALTEPPT LAYSPNRQIC PAGTTSSCTS KAVTLSSLIP EAEDSWWTGD
121 SASLDTAGIK LTVPIEKFPV TTQTFVVGCI KGDDAQSCMV TVTVQARASS VVNNVARCSY
181 GADSTLGPVK LSAEGPTTMT LVCGKDGVKV PQDNNQYCSG TTLTGCNEKS FKDILPKLTE
241 NPWQGNASSD KGATLTIKKE AFPAESKSVI IGCTGGSPEK HHCTVKLEFA GAAGSAKSAE
301 TPAPIECTAG ATKTVDAPSS GSVVFQCGDK LTISPSGEGD VFYGKECTDS RKLTTVLPGA
361 VLTAKVQQPA KGPATYTLSY DGTPEKPQVL CYKCVAEAGA PAGRNNDGSS APTPKDCKLI
421 VRVPGADGRV TSGFDPVSLT DLGTDDDDKS PGFSSTMAIM GVLVNSLGGV RVAADSGGVK
481 QTPSETGSSG GQQEAVGTTE DYVNSSAMGG GQGDSLAEDD TTSEAAEGDV DPFPVLANEG
541 KSEARGPSLE ERIEEQGTRR RYSSVQEPQA KVPCKRTQKR HRLIGAVVLA VSVAMLTAFF
601 LRRTGRRSPQ EPSGDGGGND AGNNAGNGGN EGRGYGGRGE GGAEDDRRPL HPERVNVFES
661 DPNSSSVDKL AAALEHHHHH H

SAG1-SAG2-GRA7 (SAG1 49-310 AA; SAG2 30-170 AA; GRA7 27-236 AA)

1 MHHHHHHSSG LVPRGSGMKE TAAAKFERQH MDSPDPDPPL VANQVVTCPD KKSTAAVILT
61 PTENHFTLKC PKTALTEPPT LAYSPNRQIC PAGTTSSCTS KAVTLSSLIP EAEDSWWTGD
121 SASLDTAGIK LTVPIEKFPV TTQTFVVGCI KGDDAQSCMV TVTVQARASS VVNNVARCSY
181 GADSTLGPVK LSAEGPTTMT LVCGKDGVKV PQDNNQYCSG TTLTGCNEKS FKDILPKLTE
241 NPWQGNASSD KGATLTIKKE AFPAESKSVI IGCTGGSPEK HHCTVKLEFA GAAGSAKSAE
301 TPAPIECTAG ATKTVDAPSS GSVVFQCGDK LTISPSGEGD VFYGKECTDS RKLTTVLPGA
361 VLTAKVQQPA KGPATYTLSY DGTPEKPQVL CYKCVAEAGA PAGRNNDGSS APTPKDCKLI
421 VRVPGADGRV TSGFDPVSLT DLGTDDDDKS PGFSSTMAMA TASDDELMSR IRNSDFFDGQ
481 APVDSLRPTN AGVDSKGTDD HLTTSMDKAS VESQLPRREP LETEPDEQEE VHFRKRGVRS

541 DAEVTDDNIY EEHTDRKVVP RKSEGKRSFK DLLKKLALPA VGMGASYFAA DRLVPELTEE
601 QQRGDEPLTT GQNVGTVLGF AALAAAAAFL GMGLTRTYRH FSPRKNRSRQ PALEQEVPES
661 GEDGEDARQS DPNSSSVDKL AAALEHHHHH H

SAG1-SAG2-GRA9 (SAG1 49-310 AA; SAG2 30-170 AA; GRA9 21-318 AA)

1 MHHHHHHSSG LVPRGSGMKE TAAAKFERQH MDSPDPDPPL VANQVVTCPD KKSTAAVILT
61 PTENHFTLKC PKTALTEPPT LAYSPNRQIC PAGTTSSCTS KAVTLSSLIP EAEDSWWTGD
121 SASLDTAGIK LTVPIEKFPV TTQTFVVGCI KGDDAQSCMV TVTVQARASS VVNNVARCSY
181 GADSTLGPVK LSAEGPTTMT LVCGKDGVKV PQDNNQYCSG TTLTGCNEKS FKDILPKLTE
241 NPWQGNASSD KGATLTIKKE AFPAESKSVI IGCTGGSPEK HHCTVKLEFA GAAGSAKSAE
301 TPAPIECTAG ATKTVDAPSS GSVVFQCGDK LTISPSGEGD VFYGKECTDS RKLTTVLPGA
361 VLTAKVQQPA KGPATYTLSY DGTPEKPQVL CYKCVAEAGA PAGRNNDGSS APTPKDCKLI
421 VRVPGADGRV TSGFDPVSLT DLGTDDDDKS PGFSSTMAIL DLFLGESGVY LFGKASESDV
481 ALKVPEDPVP EEPRREPEKH VDLFGEDWKQ FGGSGFGDFS KVEFENLFSQ VHEMMRRLMG
541 RGADGFGPSL LGDSPGFHFP RLRALQPKTK LEKTGTCQYV VTWAPEVTAE NVRVILHLQR
601 RQVEVQYRAA TRRDEKTEGG ESHSMSKEQS SQLMSVDPQC IMTREVVAQK LAGWTDNTHT
661 ATAGTPKKLL ISFPSPDHIK EMVKEGYLPD NALERVLAGD FEGFSRTQMC LVSGRNRTEC
721 AFAEGQEVEL EEKPLPSDSS PVTSVELPRL SQEDRGLSDP NSSSVDKLAA ALEHHHHHH

SAG1-SAG2-LDH2 (SAG1 49-310 AA; SAG2 30-170 AA; LDH2 2-326 AA)

1 MHHHHHHSSG LVPRGSGMKE TAAAKFERQH MDSPDPDPPL VANQVVTCPD KKSTAAVILT
61 PTENHFTLKC PKTALTEPPT LAYSPNRQIC PAGTTSSCTS KAVTLSSLIP EAEDSWWTGD
121 SASLDTAGIK LTVPIEKFPV TTQTFVVGCI KGDDAQSCMV TVTVQARASS VVNNVARCSY
181 GADSTLGPVK LSAEGPTTMT LVCGKDGVKV PQDNNQYCSG TTLTGCNEKS FKDILPKLTE
241 NPWQGNASSD KGATLTIKKE AFPAESKSVI IGCTGGSPEK HHCTVKLEFA GAAGSAKSAE
301 TPAPIECTAG ATKTVDAPSS GSVVFQCGDK LTISPSGEGD VFYGKECTDS RKLTTVLPGA
361 VLTAKVQQPA KGPATYTLSY DGTPEKPQVL CYKCVAEAGA PAGRNNDGSS APTPKDCKLI
421 VRVPGADGRV TSGFDPVSLT TGTVSRRKKI AMIGSGMIGG TMGYLCVLRE LADVVLFDVV
481 TGMPEGKALD DSQATSIADT NVSVTSANQY EKIAGSDVVI ITAGLTKVPG KSDKEWSRND
541 LLPFNAKIIR EVAQGVKKYC PLAFVIVVTN PLDCMVKCFH EASGLPKNMV CGMANVLDSA
601 RFRRFIADQL EISPRDIQAT VIGTHGDHML PLARYVTVNG FPLREFIKKG KMTEAKLAEI
661 VERTKKAGGE IVRLLGQGSA YYAPALSAIT MAQAFLKDEK RVLPCSVYCQ GEYGLHDMFI
721 GLPAVIGGGG IEQVIELELT HEEQECFRKS VDDVVELNKS LAALGDLGTD DDDKSPGFSS
781 TMAISDPNSS SVDKLAAALE HHHHHH

SAG1-SAG2-MAG1 (SAG1 49-310 AA; SAG2 30-170 AA; MAG1 30-452 AA)

1 MHHHHHHSSG LVPRGSGMKE TAAAKFERQH MDSPDPDPPL VANQVVTCPD KKSTAAVILT
61 PTENHFTLKC PKTALTEPPT LAYSPNRQIC PAGTTSSCTS KAVTLSSLIP EAEDSWWTGD
121 SASLDTAGIK LTVPIEKFPV TTQTFVVGCI KGDDAQSCMV TVTVQARASS VVNNVARCSY
181 GADSTLGPVK LSAEGPTTMT LVCGKDGVKV PQDNNQYCSG TTLTGCNEKS FKDILPKLTE
241 NPWQGNASSD KGATLTIKKE AFPAESKSVI IGCTGGSPEK HHCTVKLEFA GAAGSAKSAE
301 TPAPIECTAG ATKTVDAPSS GSVVFQCGDK LTISPSGEGD VFYGKECTDS RKLTTVLPGA
361 VLTAKVQQPA KGPATYTLSY DGTPEKPQVL CYKCVAEAGA PAGRNNDGSS APTPKDCKLI
421 VRVPGADGRV TSGFDPVSLT DLGTDDDDKS PGFSSTMAMS QRVPELPEVE SFDEVGTGAR
481 RSGSIATLLP QDAVLYENSE DVAVPSDSAS TPSYFHVESP SASVEAATGA VGEVVPDCEE
541 QQEQGDTTLS DHDFHSGGTE QEGLPETEVA HQHETEEQYG TEGMPPPVLP PAPVVHPRFI
601 AVPGPSVPVP FFSLPDIHPD QVVYILRVQG SGDFDISFEV GRAVKQLEAI KKAYREATGK
661 LEADELESER GPAVSPRRRL VDLIKDNQRR LRAALQKIKI QKKLEEIDDL LQLTRALKAM
721 DARLRACQDM APIEEALCHK TKAFGEMVSQ KAKEIREKAA SLSSLLGVDA VEKQLRRVEP
781 EHEDNTRVEA RVEELQKALE KAASEAKQLV GTAAGEIEEG VKADTQAVQD SSKDVLTKSQ
841 LALVEAFKAI QRALLEAKTK ELVEPTSKEA EEARQILAEQ AASDPNSSSV DKLAAALEHH
901 HHHH

SAG1-SAG2-MAG1S (SAG1 49-310 AA; SAG2 30-170 AA; MAG1S 30-222 AA)

1 MHHHHHHSSG LVPRGSGMKE TAAAKFERQH MDSPDPDPPL VANQVVTCPD KKSTAAVILT
61 PTENHFTLKC PKTALTEPPT LAYSPNRQIC PAGTTSSCTS KAVTLSSLIP EAEDSWWTGD

121 SASLDTAGIK LTVPIEKFPV TTQTFVVGCI KGDDAQSCMV TVTVQARASS VVNNVARCSY
181 GADSTLGPVK LSAEGPTTMT LVCGKDGVKV PQDNNQYCSG TTLTGCNEKS FKDILPKLTE
241 NPWQGNASSD KGATLTIKKE AFPAESKSVI IGCTGGSPEK HHCTVKLEFA GAAGSAKSAE
301 TPAPIECTAG ATKTVDAPSS GSVVFQCGDK LTISPSGEGD VFYGKECTDS RKLTTVLPGA
361 VLTAKVQQPA KGPATYTLSY DGTPEKPQVL CYKCVAEAGA PAGRNNDGSS APTPKDCKLI
421 VRVPGADGRV TSGFDPVSLT DLGTDDDDKS PGFSSTMAMS QRVPELPEVE SFDEVGTGAR
481 RSGSIATLLP QDAVLYENSE DVAVPSDSAS TPSYFHVESP SASVEAATGA VGEVVPDCEE
541 QQEQGDTTLS DHDFHSGGTE QEGLPETEVA HQHETEEQYG TEGMPPPVLP PAPVVHPRFI
601 AVPGPSVPVP FFSLPDIHPD QVVYILRVQG SGDFDISFEV GRAVKQLEAI KKSDPNSSSV
661 DKLAAALEHH HHHH

SAG1-SAG2-MIC1 (SAG1 49-310 AA; SAG2 30-170 AA; MIC1 25-456 AA)

1 MHHHHHHSSG LVPRGSGMKE TAAAKFERQH MDSPDPDPPL VANQVVTCPD KKSTAAVILT
61 PTENHFTLKC PKTALTEPPT LAYSPNRQIC PAGTTSSCTS KAVTLSSLIP EAEDSWWTGD
121 SASLDTAGIK LTVPIEKFPV TTQTFVVGCI KGDDAQSCMV TVTVQARASS VVNNVARCSY
181 GADSTLGPVK LSAEGPTTMT LVCGKDGVKV PQDNNQYCSG TTLTGCNEKS FKDILPKLTE
241 NPWQGNASSD KGATLTIKKE AFPAESKSVI IGCTGGSPEK HHCTVKLEFA GAAGSAKSAE
301 TPAPIECTAG ATKTVDAPSS GSVVFQCGDK LTISPSGEGD VFYGKECTDS RKLTTVLPGA
361 VLTAKVQQPA KGPATYTLSY DGTPEKPQVL CYKCVAEAGA PAGRNNDGSS APTPKDCKLI
421 VRVPGADGRV TSGFDPVSLT DLGTDDDDKS PGFSSTMAIA SHSHSPASGR YIQQMLDQRC
481 QEIAAELCQS GLRKMCVPSS RIVARNAVGI THQNTLQWRC FDTASLLESN QENNGVNCVD
521 DCGHTIPCPG GVHRQNSNHA TRHEILSKLV EEGVQRFCSP YQASANKYCN DKFPGTIARR
581 SKGFGNNVEV AWRCYEKASL LYSVYAECAS NCGTTWYCPG GRRGTSTELD KRHYTEEEGI
641 RQAIGSVDSP CSEVEVCLPK DENPPLCLDE SGQISRTGGG PPSQPPEMQQ PADRSDERGG
701 GKEQSPGGEA QPDHPTKGGN IDLPEKSTSP EKTPKTEIHG DSTKATLEEG QQLTLTFIST
761 KLDVAVGSCH SLVANFLDGF LKFQTGSNSA FDVVEVEEPA GPAVLTIGLG HKGRLAVVLD
821 YTRLNAALGS AAYVVEDSGC SSSEEVSFQG VGSGATLVVT TLGESPTAVS ASDPNSSSVD
881 KLAAALEHHH HHH

SAG1-SAG2-MIC1ex2 (SAG1 49-310 AA; SAG2 30-170 AA; MIC1ex2 25-182 AA)

1 MHHHHHHSSG LVPRGSGMKE TAAAKFERQH MDSPDPDPPL VANQVVTCPD KKSTAAVILT
61 PTENHFTLKC PKTALTEPPT LAYSPNRQIC PAGTTSSCTS KAVTLSSLIP EAEDSWWTGD
121 SASLDTAGIK LTVPIEKFPV TTQTFVVGCI KGDDAQSCMV TVTVQARASS VVNNVARCSY
181 GADSTLGPVK LSAEGPTTMT LVCGKDGVKV PQDNNQYCSG TTLTGCNEKS FKDILPKLTE
241 NPWQGNASSD KGATLTIKKE AFPAESKSVI IGCTGGSPEK HHCTVKLEFA GAAGSAKSAE
301 TPAPIECTAG ATKTVDAPSS GSVVFQCGDK LTISPSGEGD VFYGKECTDS RKLTTVLPGA
361 VLTAKVQQPA KGPATYTLSY DGTPEKPQVL CYKCVAEAGA PAGRNNDGSS APTPKDCKLI
421 VRVPGADGRV TSGFDPVSLT ASHSHSPASG RYIQQMLDQR CQEIAAELCQ SGLRKMCVPS
481 SRIVARNAVG ITHQNTLQWR CFDTASLLES NQENNGVNCV DDCGHTIPCP GGVHRQNSNH
541 ATRHEILSKL VEEGVQRFCS PYQASANKYC NDKFPGTIAR RSKGFGNNVE VAWRCYEKDL
601 GTDDDDKSPG FSSTMAISDP NSSSVDKLAA ALEHHHHHH

SAG1-SAG2-MIC3 (SAG1 49-310 AA; SAG2 30-170 AA; MIC3 67-359 AA)

1 MHHHHHHSSG LVPRGSGMKE TAAAKFERQH MDSPDPDPPL VANQVVTCPD KKSTAAVILT
61 PTENHFTLKC PKTALTEPPT LAYSPNRQIC PAGTTSSCTS KAVTLSSLIP EAEDSWWTGD
121 SASLDTAGIK LTVPIEKFPV TTQTFVVGCI KGDDAQSCMV TVTVQARASS VVNNVARCSY
181 GADSTLGPVK LSAEGPTTMT LVCGKDGVKV PQDNNQYCSG TTLTGCNEKS FKDILPKLTE
241 NPWQGNASSD KGATLTIKKE AFPAESKSVI IGCTGGSPEK HHCTVKLEFA GAAGSAKSAE
301 TPAPIECTAG ATKTVDAPSS GSVVFQCGDK LTISPSGEGD VFYGKECTDS RKLTTVLPGA
361 VLTAKVQQPA KGPATYTLSY DGTPEKPQVL CYKCVAEAGA PAGRNNDGSS APTPKDCKLI
421 VRVPGADGRV TSGFDPVSLT SPSKQETQLC AISSEGKPCR NRQLHTDNGY FIGASCPKSA
481 CCSKTMCGPG GCGEFCSSNW IFCSSSLIYH PDKSYGGDCS CEKQGHRCDK NAECVENLDA
541 GGGVHCKCKD GFVGTGLTCS EDPCSKRGNA KCGPNGTCIV VDSVSYTCTC GDGETLVNLP
601 EGGQGCKRTG CHAFRENCSP GRCIDDASHE NGYTCECPTG YSREVTSKAE ESCVEGVEVT
661 LAEKCEKEFG ISASSCKCDN GYSGSASATS HHGKGESGSE GSLSEKMNIV FKCPSGYHPR
721 YHAHTVTCEK IKQDLGTDDD DKSPGFSSTM AISDPNSSSV DKLAAALEHH HHHH

SAG1-SAG2-P35 (SAG1 49-310 AA; SAG2 30-170 AA; P35 26-377 AA)

```
  1 MHHHHHHSSG LVPRGSGMKE TAAAKFERQH MDSPDPDPPL VANQVVTCPD KKSTAAVILT
 61 PTENHFTLKC PKTALTEPPT LAYSPNRQIC PAGTTSSCTS KAVTLSSLIP EAEDSWWTGD
121 SASLDTAGIK LTVPIEKFPV TTQTFVVGCI KGDDAQSCMV TVTVQARASS VVNNVARCSY
181 GADSTLGPVK LSAEGPTTMT LVCGKDGVKV PQDNNQYCSG TTLTGCNEKS FKDILPKLTE
241 NPWQGNASSD KGATLTIKKE AFPAESKSVI IGCTGGSPEK HHCTVKLEFA GAAGSAKSAE
301 TPAPIECTAG ATKTVDAPSS GSVVFQCGDK LTISPSGEGD VFYGKECTDS RKLTTVLPGA
361 VLTAKVQQPA KGPATYTLSY DGTPEKPQVL CYKCVAEAGA PAGRNNDGSS APTPKDCKLI
421 VRVPGADGRV TSGFDPVSLT DLGTDDDDKS PGFSSTMAIG PLSYHPSSYG ASYPNPSNPL
481 HGMPKPENPV RPPPPGFHPS VIPNPPYPLG TPAGMPQPEV PPLQHPPPTG SPPAAAPQPP
541 YPVGTPVMPQ PEIPPVHRPP PPGFRPEVAP VPPYPVGTPT GMPQPEIPAV HHPFPYVTTT
601 TTAAPRVLVY KIPYGGAAPP RAPPVPPRMG PSDISTHVRG AIRRQPGTTT TTTSRKLLFR
661 TAVVAAMAAA LITLFRQRPV FMEGVRMFPN LHMPQPEIPA VHHPFPYVTT TTTAAPRVLV
721 YKIPYGGAAP PRAPPVPPRM GPSDISTHVR GAIRRQPGTT TTTTSRKLLF RTAVVAAMAA
781 ALITLFRQRP VFMEGVRMFP NLHYRFTVTT QKSDPNSSSV DKLAAALEHH HHHH
```

SAG1-SAG2-P35S (SAG1 49-310 AA; SAG2 30-170 AA; P35S 26-170 AA)

```
  1 MHHHHHHSSG LVPRGSGMKE TAAAKFERQH MDSPDPDPPL VANQVVTCPD KKSTAAVILT
 61 PTENHFTLKC PKTALTEPPT LAYSPNRQIC PAGTTSSCTS KAVTLSSLIP EAEDSWWTGD
121 SASLDTAGIK LTVPIEKFPV TTQTFVVGCI KGDDAQSCMV TVTVQARASS VVNNVARCSY
181 GADSTLGPVK LSAEGPTTMT LVCGKDGVKV PQDNNQYCSG TTLTGCNEKS FKDILPKLTE
241 NPWQGNASSD KGATLTIKKE AFPAESKSVI IGCTGGSPEK HHCTVKLEFA GAAGSAKSAE
301 TPAPIECTAG ATKTVDAPSS GSVVFQCGDK LTISPSGEGD VFYGKECTDS RKLTTVLPGA
361 VLTAKVQQPA KGPATYTLSY DGTPEKPQVL CYKCVAEAGA PAGRNNDGSS APTPKDCKLI
421 VRVPGADGRV TSGFDPVSLT GPLSYHPSSY GASYPNPSNP LHGMPKPENP VRPPPPGFHP
481 SVIPNPPYPL GTPAGMPQPE VPPLQHPPPT GSPPAAAPQP PYPVGTPVMP QPEIPPVHRP
541 PPPGFRPEVA PVPPYPVGTP TGMPQPEIPA VHHPFPYVTT TTTAADLGTD DDDKSPGFSS
601 TMAISDPNSS SVDKLAAALE HHHHHH
```

SAG1-SAG2-ROP1 (SAG1 49-310 AA; SAG2 30-170 AA; ROP1 85-396 AA)

```
  1 MHHHHHHSSG LVPRGSGMKE TAAAKFERQH MDSPDPDPPL VANQVVTCPD KKSTAAVILT
 61 PTENHFTLKC PKTALTEPPT LAYSPNRQIC PAGTTSSCTS KAVTLSSLIP EAEDSWWTGD
121 SASLDTAGIK LTVPIEKFPV TTQTFVVGCI KGDDAQSCMV TVTVQARASS VVNNVARCSY
181 GADSTLGPVK LSAEGPTTMT LVCGKDGVKV PQDNNQYCSG TTLTGCNEKS FKDILPKLTE
241 NPWQGNASSD KGATLTIKKE AFPAESKSVI IGCTGGSPEK HHCTVKLEFA GAAGSAKSAE
301 TPAPIECTAG ATKTVDAPSS GSVVFQCGDK LTISPSGEGD VFYGKECTDS RKLTTVLPGA
361 VLTAKVQQPA KGPATYTLSY DGTPEKPQVL CYKCVAEAGA PAGRNNDGSS APTPKDCKLI
421 VRVPGADGRV TSGFDPVSLT PVRGPDQVPA RGEAALVTEE TPAQQPAVAL GSAEGEGTST
481 TESASENSED DDTFHDALQE LPEDGLEVRP PNAQELPPPN VQELPPPTEQ ELPPSTEQEL
541 PPPVGEGQRL QVPGEHGPQG PPYDDQQLLL EPTEEQQEGP QEPLPPPPPP TRGEQPEGQQ
601 PQGPVRQNFF RRALGAARSR FGGARRHVSG VFRRVRGGLN RIVGGVRSGF RRAREGVVGG
661 VRRLTSGASL GLRRVGEGLR RSFYRVRGAV SSGRRRAADG ASNVRERFVA AGGRVRDAFG
721 AGLTRLRRRG RTNGEEGRPL LGEGREQDDG SQDLGTDDDD KSPGFSSTMA ISDPNSSSVD
781 KLAAALEHHH HHH
```
