# Supplementary material for: The Development of Toxoplasma gondii Recombinant Trivalent Chimeric Proteins as an Alternative to Toxoplasma Lysate Antigen (TLA) in Enzyme-Linked Immunosorbent Assay (ELISA) for the Detection of Immunoglobulin G (IgG) in Small Ruminants
Source: Int J Mol Sci. 2024 Apr 16;25(8):4384. doi: 10.3390/ijms25084384 (PMC11049947; doi:10.3390/ijms25084384)

Gel A

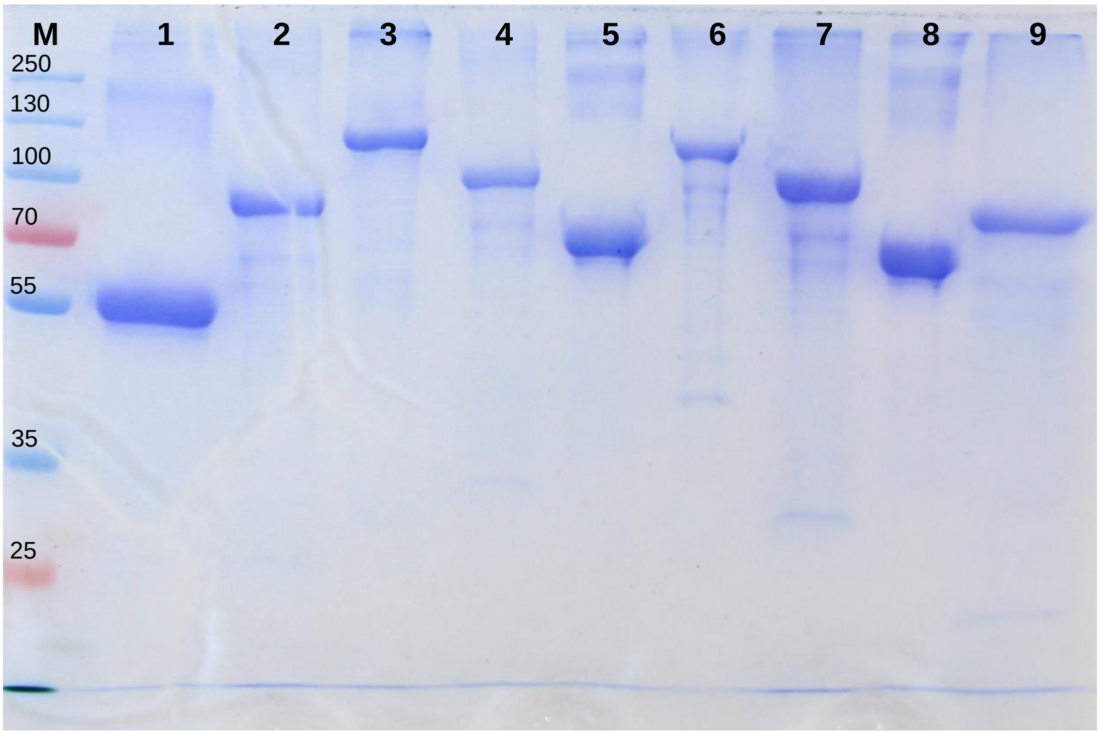

Gel B

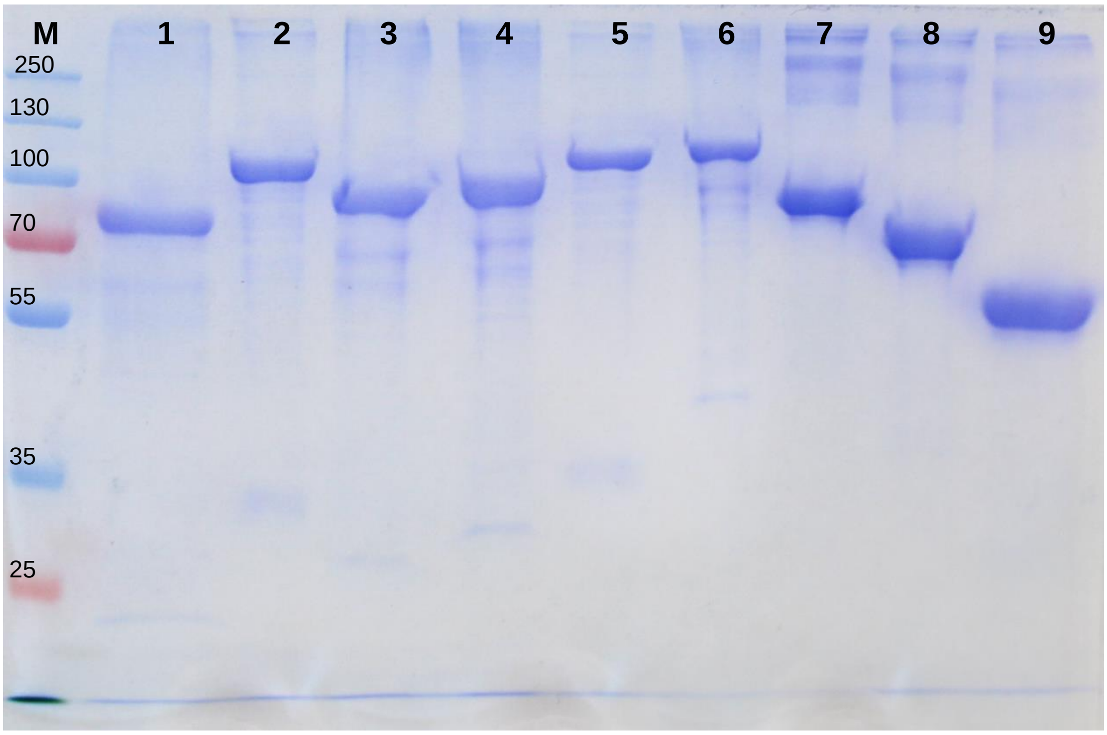

Figure S7. Coomassie blue-stained SDS-PAGE (10% polyacrylamide gel) separation of purified recombinant chimeric proteins.

Gel A lines:

M – PageRuler™ Plus Prestained Protein Ladder (Thermo Fisher Scientific, Waltham, MA, USA)

1 – SAG1-SAG2

2 – SAG1-SAG2-MAG1S

3 – SAG1-SAG2-MIC1

4 – SAG1-SAG2-GRA9

5 – SAG1-SAG2-P35S
6 – SAG1-SAG2-P35
7 – SAG1-SAG2-GRA7
8 – SAG1-SAG2-GRA2
9 – SAG1-SAG2-GRA1

Gel B lines:
M – PageRuler™ Plus Prestained Protein Ladder (Thermo Fisher Scientific, Waltham, MA, USA)
1 – SAG1-SAG2-GRA6
2 – SAG1-SAG2-MAG1
3 – SAG1-SAG2-ROP1
4 – SAG1-SAG2-LDH2
5 – SAG1-SAG2-AMA1S
6 – SAG1-SAG2-AMA1C
7 – SAG1-SAG2-MIC3
8 – SAG1-SAG2-MIC1ex2
9 – SAG1-SAG2-GRA5

Additionally, we attach uncropped photos of the gels.

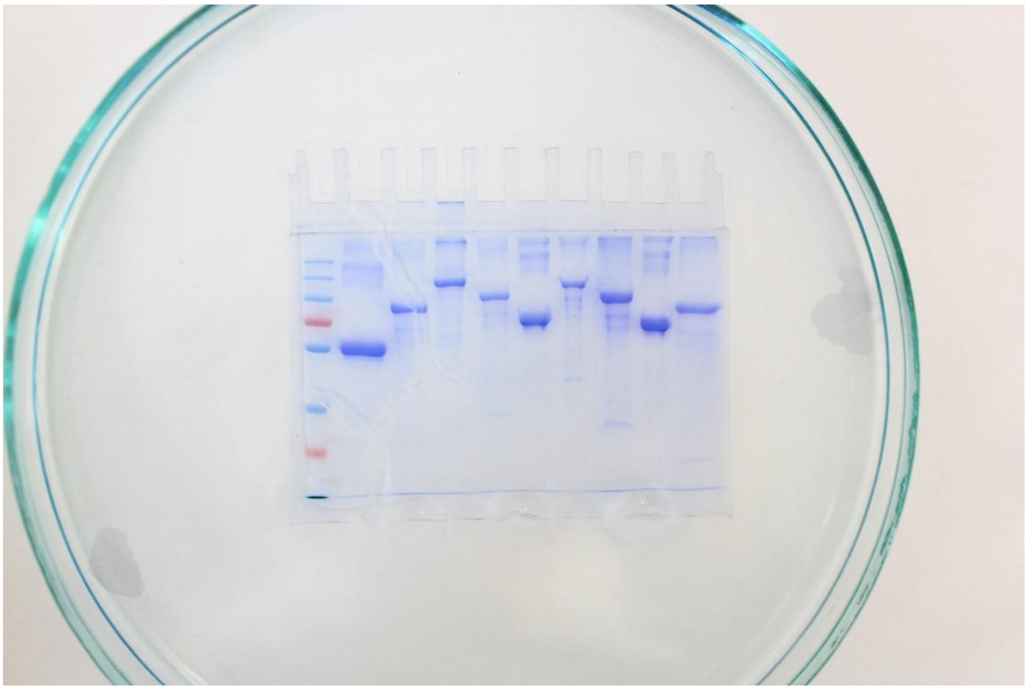

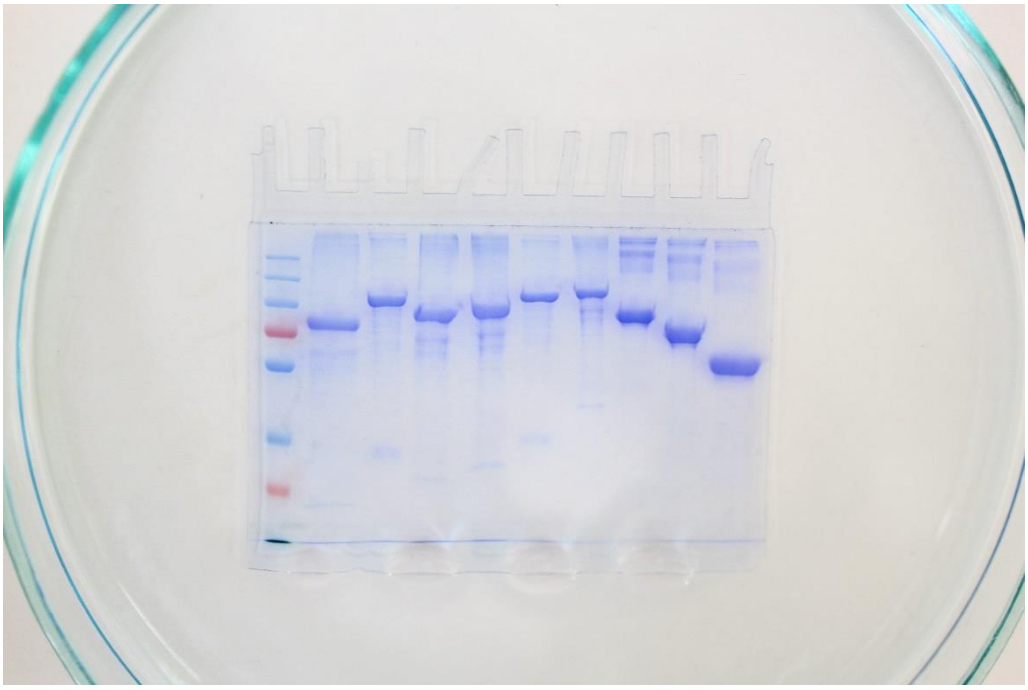

Supplement: Supplementary file 1 [file ijms-25-04384-s001.zip › Figure S7.pdf]
