# Supplementary material for: The Development of Toxoplasma gondii Recombinant Trivalent Chimeric Proteins as an Alternative to Toxoplasma Lysate Antigen (TLA) in Enzyme-Linked Immunosorbent Assay (ELISA) for the Detection of Immunoglobulin G (IgG) in Small Ruminants
Source: Int J Mol Sci. 2024 Apr 16;25(8):4384. doi: 10.3390/ijms25084384 (PMC11049947; doi:10.3390/ijms25084384)
Supplement: Supplementary file 1 [file ijms-25-04384-s001.zip › Table S1.pdf]

Table S1. Characteristics of recombinant plasmids containing fusion genes encoding recombinant chimeric proteins.

Recombinant plasmid	Plasmid size [bp]	Gene fragment	Additional gene fragment	Encoding amino acid residues	GenBank Accession No.	Nucleotide
pET30/SAG1-SAG2-AMA1	8163	*sag1* (amino acid residues from 49-310); GeneBank Accession No. S76248.1; nucleotide from 453-1244 *sag2* (amino acid from 30-170); GeneBank Accession No. M33572.1; nucleotide from 269-691*/ 269-692**/ 269-693***	*ama1*	from 67-568	XM_002364813.1	727-2232
pET30/SAG1-SAG2-AMA1S	7908		*ama1*	from 67-483	XM_002364813.1	728-1979
pET30/SAG1-SAG2-GRA1	7158		*gra1*	from 24-190	M26007.1	681-1181
pET30/SAG1-SAG2-GRA2	7062		*gra2*	from 51-185	M99392.1	1128-1532
pET30/SAG1-SAG2-GRA5	6942		*gra5*	from 26-120	L06091.1	391-675
pET30/SAG1-SAG2-GRA6	7257		*gra6*	from 30-228	L33814.1	506-1106
pET30/SAG1-SAG2-GRA7	7287		*gra7*	from 27-236	Y13863.1	156-785
pET30/SAG1-SAG2-GRA9	7551		*gra9*	from 21-318	AY371455.1	2148-2498 and 3116-3658
pET30/SAG1-SAG2-LDH2	7632		*ldh2*	from 2-326	XM_002368447.2	909-1884
pET30/SAG1-SAG2-MAG1	7926		*mag1*	from 30-452	XM_002365659.1	374-1643
pET30/SAG1-SAG2-MAG1S	7236		*mag1s*	from 30-222	XM_002365659.1	374-954
pET30/SAG1-SAG2-MIC1	7953		*mic1*	from 25-456	Z71786.1	216-217, 537-1009, 1401-1479 and 1742-2483
pET30/SAG1-SAG2-MIC1S	7131		*mic1ex2*	from 25-182	Z71786.1	217 and 537-1010
pET30/SAG1-SAG2-MIC3	7536		*mic3*	from 67-359	AJ132530.1	894-1772
pET30/SAG1-SAG2-P35	7716		*p35*	from 26-377	AF310261.1	120-1178
pET30/SAG1-SAG2-P35S	7092		*p35*	from 26-170	AF310261.1	121-555
pET30/SAG1-SAG2-ROP1	7593		*rop1*	from 85-396	M71274.1	453-1388
pET30/SAG1-SAG2	6657	*sag1*	-	from 49-310	S76248.1	453-1244
		sag2	-	from 30-170	M33572.1	269-692

Explanation of symbols: * - in case of pET30/SAG1-SAG2-AMA1N, pET30/SAG1-SAG2-LDH2, pET30/SAG1-SAG2-MIC3, pET30/SAG1-SAG2-ROP1; ** - in case of pET30/SAG1-SAG2-AMA1, pET30/SAG1-SAG2-GRA1, pET30/SAG1-SAG2-GRA5, pET30/SAG1-SAG2-GRA6, pET30/SAG1-SAG2-GRA7, pET30/SAG1-SAG2-GRA9, pET30/SAG1-SAG2-MAG1, pET30/SAG1-SAG2-MAG1S, pET30/SAG1-SAG2-MIC1, pET30/SAG1-SAG2-MIC1S, pET30/SAG1-SAG2-P35; *** - in case of pET30/SAG1-SAG2-GRA2, pET30/SAG1-SAG2-P35S
