# Supplementary material for: The Development of Toxoplasma gondii Recombinant Trivalent Chimeric Proteins as an Alternative to Toxoplasma Lysate Antigen (TLA) in Enzyme-Linked Immunosorbent Assay (ELISA) for the Detection of Immunoglobulin G (IgG) in Small Ruminants
Source: Int J Mol Sci. 2024 Apr 16;25(8):4384. doi: 10.3390/ijms25084384 (PMC11049947; doi:10.3390/ijms25084384)
Supplement: Supplementary file 1 [file ijms-25-04384-s001.zip › Table S2.pdf]

Table S2. Characteristics of the single recombinant proteins.

Recombinant protein	Amino acid residues	Mw [kDa]	pI	Reference
AMA1	from 67-569	61.29	5.97	[1]
AMA1C	from 287-569	35.91	5.41	[1]
AMA1N	from 67-287	32.07	8.41	[1]
BAG1	from 1-229	30.60	6.45	[2]
BSR4	from 33-356	40.40	5.90	[2]
GRA1	from 24-190	24.09	4.79	[3]
GRA2	from 51-185	23.49	6.91	[4]
GRA4	from 20-279	31.87	7.22	[2]
GRA5	from 26-94	15.90	6.45	[5]
GRA6	from 30-231	27.24	6.00	[6]
GRA7	from 18-236	32.60	5.77	[3]
GRA9	from 21-318	38.86	5.92	[2]
LDH1	from 1-329	41.70	5.99	[2]
LDH2	from 1-326	41.50	6.25	[2]
MAG1	from 30-222	23.81	4.94	[7]
MIC1	from 25-456	52.00	5.89	[8]
MIC1ex2	from 25-182	24.50	7.79	[8]
MIC1ex3,4	from 183-456	35.90	4.97	[8]
MIC3	from 67-359	36.52	6.45	[2]
P35	from 26-170	21.30	6.85	[6]
ROP1	from 85-396	39.11	6.66	[4]
SAG1	from 49-313	33.42	6.75	[3]
SAG2	from 30-170	20.50	6.88	[6]
SAG4	from 26-150	19.24	7.61	[2]

Reference

1. Ferra, B.; Holec-Gąsior, L.; Gatkowska, J.; Dziadek, B.; Dzitko, K. *Toxoplasma Gondii* Recombinant Antigen AMA1: Diagnostic Utility of Protein Fragments for the Detection of IgG and IgM Antibodies. *Pathogens* **2020**, *9*, doi:10.3390/pathogens9010043.
2. Holec-Gasior, L.; Ferra, B.; Hiszczyńska-Sawicka, E.; Kur, J. The Optimal Mixture of *Toxoplasma Gondii* Recombinant Antigens (GRA1, P22, ROP1) for Diagnosis of Ovine Toxoplasmosis. *Vet Parasitol* **2014**, *206*, 146–152, doi:10.1016/j.vetpar.2014.09.018.
3. Hiszczyńska-Sawicka, E.; Brillowska-Dąbrowska, A.; Dąabrowski, S.; Pietkiewicz, H.; Myjak, P.; Kur, J. High Yield Expression and Single-Step Purification of *Toxoplasma Gondii* SAG1, GRA1, and GRA7 Antigens in *Escherichia Coli*. *Protein Expr Purif* **2003**, *27*, 150–157, doi:10.1016/S1046-5928(02)00593-4.
4. Holec-Gasior, L.; Kur, J.; Hiszczyńska-Sawicka, E. GRA2 and ROP1 Recombinant Antigens as Potential Markers for Detection of *Toxoplasma Gondii*-Specific Immunoglobulin G in Humans with Acute Toxoplasmosis. *Clin Vaccine Immunol* **2009**, *16*, 510–514, doi:10.1128/CVI.00341-08.
5. Holec-Gasior, L.; Kur, J. *Toxoplasma Gondii*: Recombinant GRA5 Antigen for Detection of Immunoglobulin G Antibodies Using Enzyme-Linked Immunosorbent Assay. *Exp Parasitol* **2010**, *124*, 272–278, doi:10.1016/J.EXPPARA.2009.10.010.
6. Hiszczyńska-Sawicka, E.; Kur, J.; Pietkiewicz, H.; Holec, L.; Gąsior, A.; Myjak, P. Efficient Production of the *Toxoplasma Gondii* GRA6, P35 and SAG2 Recombinant Antigens and Their Applications in the Serodiagnosis of Toxoplasmosis. *Acta Parasitol* **2005**, *50*, 249–254.

7. Holec, L.; Hiszczyńska-Sawicka, E.; Gasior, A.; Brillowska-Dabrowska, A.; Kur, J. Use of MAG1 Recombinant Antigen for Diagnosis of *Toxoplasma Gondii* Infection in Humans. *Clin Vaccine Immunol* **2007**, *14*, 220–225, doi:10.1128/CVI.00419-06.
8. Holec, L.; Gąsior, A.; Brillowska-Dąbrowska, A.; Kur, J. *Toxoplasma Gondii*: Enzyme-Linked Immunosorbent Assay Using Different Fragments of Recombinant Microneme Protein 1 (MIC1) for Detection of Immunoglobulin G Antibodies. *Exp Parasitol* **2008**, *119*, 1–6, doi:10.1016/J.EXPPARA.2007.12.002.
